# 1-Chloro­meth­yl-3-nitro­benzene

**DOI:** 10.1107/S1600536810005076

**Published:** 2010-02-13

**Authors:** Muhammad Athar Abbasi, Muhammad Jahangir, Mehmet Akkurt, Aziz-ur-Rehman, Islam Ullah Khan, Shahzad Sharif

**Affiliations:** a Department of Chemistry, Government College University, Lahore 54000, Pakistan; b Department of Physics, Faculty of Arts and Sciences, Erciyes University, 38039 Kayseri, Turkey

## Abstract

In the title mol­ecule, C_7_H_6_ClNO_2_, the plane of the nitro group and the direction of the chloro­methyl group are twisted away from the benzene ring, forming dihedral angles of 8.2 (3) and 67.55 (12)°, respectively. In the crystal structure, weak inter­molecular C—H⋯O inter­actions link the mol­ecules into corrugated sheets parallel to the *bc* plane.

## Related literature

For the characteristics of nitro­aromatic compounds, see: Moreno *et al.* (1986 ▶). For details of the synthesis, see: Livermore & Sealock (1947 ▶). For reference bond lengths, see: Allen *et al.* (1987 ▶).
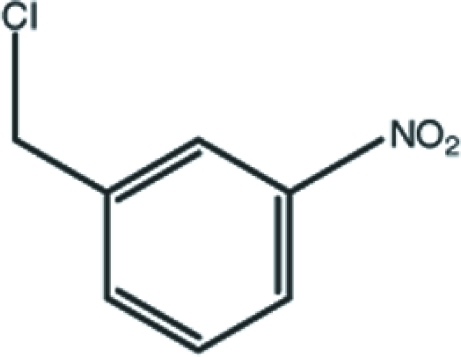


## Experimental

### 

#### Crystal data

C_7_H_6_ClNO_2_
*M* *_r_* = 171.58
Monoclinic, 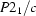

*a* = 12.1219 (10) Å
*b* = 4.5104 (4) Å
*c* = 15.1219 (11) Å
β = 112.709 (2)°
*V* = 762.69 (11) Å^3^
*Z* = 4
Mo *K*α radiation
μ = 0.44 mm^−1^
*T* = 296 K
0.34 × 0.18 × 0.11 mm

#### Data collection

Bruker Kappa APEXII CCD area-detector diffractometer
8475 measured reflections
1903 independent reflections
1350 reflections with *I* > 2σ(*I*)
*R*_int_ = 0.023

#### Refinement

*R*[*F* ^2^ > 2σ(*F* ^2^)] = 0.041
*wR*(*F* ^2^) = 0.116
*S* = 1.03
1903 reflections
100 parameters
H-atom parameters constrained
Δρ_max_ = 0.25 e Å^−3^
Δρ_min_ = −0.26 e Å^−3^

### 

Data collection: *APEX2* (Bruker, 2007 ▶); cell refinement: *SAINT* (Bruker, 2007 ▶); data reduction: *SAINT*; program(s) used to solve structure: *SHELXS97* (Sheldrick, 2008 ▶); program(s) used to refine structure: *SHELXL97* (Sheldrick, 2008 ▶); molecular graphics: *ORTEP-3 for Windows* (Farrugia, 1997 ▶); software used to prepare material for publication: *WinGX* (Farrugia, 1999 ▶) and *PLATON* (Spek, 2009 ▶).

## Supplementary Material

Crystal structure: contains datablocks global, I. DOI: 10.1107/S1600536810005076/cv2694sup1.cif

Structure factors: contains datablocks I. DOI: 10.1107/S1600536810005076/cv2694Isup2.hkl

Additional supplementary materials:  crystallographic information; 3D view; checkCIF report

## Figures and Tables

**Table 1:** Hydrogen-bond geometry (Å, °)

*D*—H⋯*A*	*D*—H	H⋯*A*	*D*⋯*A*	*D*—H⋯*A*
C2—H2⋯O1^i^	0.93	2.67	3.583 (3)	166
C6—H6⋯O2^ii^	0.93	2.67	3.374 (3)	133

Symmetry codes: (i) 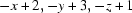; (ii) 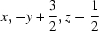.

**Fig. 1.**
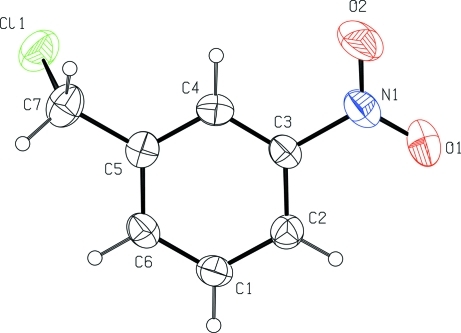
The title molecule with the atom numbering scheme. Displacement ellipsoids for non-H atoms are drawn at the 30% probability level.

## supplementary crystallographic information

### Comment

The irreversible binding of the reductive intermediates of nitroaromatic compounds to protein and DNA is thought to be responsible for the carcinogenicity and mutagenicity of this class of compounds. Several studies revealed that some nitro radical metabolites with special features are expected to decompose to form neutral carbon-centered free radicals with not net reduction of the nitro group occuring. The radicals anions of *p*-and *o*-nitrobenzyl chloride are known to expel chloride to form the corresponding carbon-centered nitrobenzyl radicals with rate constants of 1 × 10^4^ and 4 × 10^3 ^s^-1^. Such species are highly reactive and could account for the unusual cytotoxicity of these nitrocompounds (Moreno *et al.*, 1986). This structural report on 1-(chloromethyl)-3-nitrobenzene (*m*-nitrobenzyl chloride) might be helpful to carry out such studies on these nitroaromatic compounds in future.

The molecule of the title compound has normal bond lengths (Allen *et al.*, 1987). The benzene ring (C1-C6) forms dihedral angles of 8.2 (3) and 67.55 (12)°, with the plane of  the nitro group (N1/O1/O2) and with the direction of the chloromethyl group (C7/Cl1), respectively.

In the crystal structure, there is no classic hydrogen bonds. Weak intermolecular C—H···O interactions (Table 1) link molecules into corrugated sheets parallel to *bc* plane.

### Experimental

1-(Chloromethyl)-3-nitrobenzene (*m*-nitrobenzyl chloride) was prepared from the *m*-nitrobenzyl alcohol, 5 g; being refluxed with 25 ml of concentrated hydrochloric acid on a boiling water bath for 1.5 h. The ether was washed with water and sodium carbonate and dried with sodium sulfate. Evaporation of ether yielded an oily residue which crystallized on cooling. A 70 per cent yield of the crude compound was obtained. Recrystallization from petroleum ether gave a product melting at 317-319 K (Livermore and Sealock, 1947). *m*-Nitrobenzyl alcohol was purchased from Sigma Aldrich while all other chemicals involved were obtained from Merk, Germany.

### Refinement

H atoms were geometrically positioned (C—H = 0.93-0.97 Å), and treated using a riding model, with U_iso_(H) = 1.2U_eq_(C).

### Crystal data

**Table:**

C_7_H_6_ClNO_2_	*F*(000) = 352
*M**_r_* = 171.58	*D*_x_ = 1.494 Mg m^−^^3^
Monoclinic, *P*2_1_/*c*	Mo *K*α radiation, λ = 0.71073 Å
Hall symbol: -P 2ybc	Cell parameters from 2610 reflections
*a* = 12.1219 (10) Å	θ = 2.8–26.9°
*b* = 4.5104 (4) Å	µ = 0.44 mm^−^^1^
*c* = 15.1219 (11) Å	*T* = 296 K
β = 112.709 (2)°	Slab, pale yellow
*V* = 762.69 (11) Å^3^	0.34 × 0.18 × 0.11 mm
*Z* = 4	

### Data collection

**Table:**

Bruker Kappa APEXII CCD area-detector diffractometer	1350 reflections with *I* > 2σ(*I*)
Radiation source: sealed tube	*R*_int_ = 0.023
graphite	θ_max_ = 28.4°, θ_min_ = 1.8°
φ and ω scans	*h* = −16→16
8475 measured reflections	*k* = −6→5
1903 independent reflections	*l* = −19→20

### Refinement

**Table:**

Refinement on *F*^2^	Primary atom site location: structure-invariant direct methods
Least-squares matrix: full	Secondary atom site location: difference Fourier map
*R*[*F*^2^ > 2σ(*F*^2^)] = 0.041	Hydrogen site location: inferred from neighbouring sites
*wR*(*F*^2^) = 0.116	H-atom parameters constrained
*S* = 1.03	*w* = 1/[σ^2^(*F*_o_^2^) + (0.0443*P*)^2^ + 0.2569*P*] where *P* = (*F*_o_^2^ + 2*F*_c_^2^)/3
1903 reflections	(Δ/σ)_max_ < 0.001
100 parameters	Δρ_max_ = 0.25 e Å^−^^3^
0 restraints	Δρ_min_ = −0.26 e Å^−^^3^

### Special details

**Table:**

Geometry. Bond distances, angles etc. have been calculated using the rounded fractional coordinates. All su's are estimated from the variances of the (full) variance-covariance matrix. The cell esds are taken into account in the estimation of distances, angles and torsion angles
Refinement. Refinement on *F*^2^ for ALL reflections except those flagged by the user for potential systematic errors. Weighted *R*-factors wR and all goodnesses of fit *S* are based on *F*^2^, conventional *R*-factors *R* are based on *F*, with *F* set to zero for negative *F*^2^. The observed criterion of *F*^2^ > σ(*F*^2^) is used only for calculating -*R*-factor-obs etc. and is not relevant to the choice of reflections for refinement. *R*-factors based on *F*^2^ are statistically about twice as large as those based on *F*, and *R*-factors based on ALL data will be even larger.

### Fractional atomic coordinates and isotropic or equivalent isotropic displacement parameters (Å^2^)

**Table:**

	*x*	*y*	*z*	*U*_iso_*/*U*_eq_	
Cl1	0.46056 (5)	0.88735 (16)	0.10489 (5)	0.0856 (2)	
O1	0.91285 (17)	1.2499 (5)	0.51307 (11)	0.0999 (7)	
O2	0.78454 (18)	0.9074 (5)	0.49378 (12)	0.1009 (8)	
N1	0.83972 (16)	1.0739 (5)	0.46346 (11)	0.0666 (6)	
C1	0.86599 (17)	1.2052 (5)	0.23036 (13)	0.0593 (6)	
C2	0.88908 (16)	1.2205 (5)	0.32703 (13)	0.0559 (6)	
C3	0.81640 (15)	1.0604 (4)	0.36006 (11)	0.0499 (5)	
C4	0.72367 (16)	0.8898 (4)	0.30166 (13)	0.0540 (6)	
C5	0.70089 (15)	0.8763 (4)	0.20455 (13)	0.0526 (6)	
C6	0.77357 (17)	1.0351 (4)	0.17013 (12)	0.0559 (6)	
C7	0.60014 (19)	0.6926 (5)	0.13890 (17)	0.0738 (8)	
H1	0.91350	1.31120	0.20570	0.0710*	
H2	0.95160	1.33500	0.36830	0.0670*	
H4	0.67650	0.78420	0.32670	0.0650*	
H6	0.75960	1.02650	0.10520	0.0670*	
H7A	0.59560	0.50880	0.17070	0.0890*	
H7B	0.61490	0.64360	0.08200	0.0890*	

### Atomic displacement parameters (Å^2^)

**Table:**

	*U*^11^	*U*^22^	*U*^33^	*U*^12^	*U*^13^	*U*^23^
Cl1	0.0538 (3)	0.1001 (5)	0.0947 (4)	−0.0057 (3)	0.0195 (3)	−0.0148 (3)
O1	0.1089 (13)	0.1307 (16)	0.0585 (9)	−0.0134 (13)	0.0306 (9)	−0.0266 (10)
O2	0.1138 (14)	0.1320 (17)	0.0669 (10)	0.0001 (12)	0.0459 (10)	0.0313 (10)
N1	0.0684 (10)	0.0841 (13)	0.0505 (9)	0.0193 (10)	0.0265 (8)	0.0092 (9)
C1	0.0587 (10)	0.0689 (12)	0.0572 (10)	−0.0008 (9)	0.0300 (9)	0.0060 (9)
C2	0.0500 (9)	0.0607 (11)	0.0548 (10)	0.0006 (9)	0.0179 (8)	−0.0025 (9)
C3	0.0518 (9)	0.0558 (11)	0.0436 (8)	0.0127 (8)	0.0200 (7)	0.0054 (7)
C4	0.0546 (9)	0.0498 (10)	0.0624 (10)	0.0059 (8)	0.0278 (8)	0.0097 (8)
C5	0.0517 (9)	0.0445 (10)	0.0583 (10)	0.0084 (8)	0.0175 (8)	−0.0027 (8)
C6	0.0593 (10)	0.0640 (12)	0.0471 (9)	0.0104 (9)	0.0236 (8)	0.0004 (8)
C7	0.0683 (13)	0.0587 (12)	0.0863 (15)	0.0003 (10)	0.0210 (11)	−0.0148 (11)

### Geometric parameters (Å, °)

**Table:**

Cl1—C7	1.796 (3)	C5—C6	1.384 (3)
O1—N1	1.211 (3)	C5—C7	1.493 (3)
O2—N1	1.208 (3)	C1—H1	0.9300
N1—C3	1.479 (2)	C2—H2	0.9300
C1—C2	1.380 (3)	C4—H4	0.9300
C1—C6	1.373 (3)	C6—H6	0.9300
C2—C3	1.374 (3)	C7—H7A	0.9700
C3—C4	1.367 (3)	C7—H7B	0.9700
C4—C5	1.387 (3)		
			
Cl1···H4^i^	2.8900	C6···C7^vi^	3.560 (3)
O1···N1^ii^	3.230 (3)	C6···O2^viii^	3.374 (3)
O1···O1^ii^	3.217 (3)	C7···C6^vii^	3.560 (3)
O1···O1^iii^	3.218 (3)	C1···H1^ix^	3.0400
O1···C2^ii^	3.410 (3)	C5···H7A^vi^	3.0900
O1···C3^ii^	3.399 (3)	C6···H7A^vi^	3.0400
O2···C6^iv^	3.374 (3)	H1···C1^x^	3.0400
O1···H2	2.4400	H2···O1	2.4400
O1···H6^v^	2.9000	H2···O1^iii^	2.6700
O1···H2^iii^	2.6700	H4···O2	2.4200
O2···H4	2.4200	H4···H7A	2.5100
O2···H6^iv^	2.6700	H4···Cl1^xi^	2.8900
O2···H7B^iv^	2.8600	H6···H7B	2.3900
N1···O1^ii^	3.230 (3)	H6···O1^xii^	2.9000
C1···C5^vi^	3.566 (3)	H6···O2^viii^	2.6700
C2···C4^vi^	3.562 (3)	H7A···C5^vii^	3.0900
C2···O1^ii^	3.410 (3)	H7A···C6^vii^	3.0400
C3···O1^ii^	3.399 (3)	H7A···H4	2.5100
C4···C2^vii^	3.562 (3)	H7B···H6	2.3900
C5···C1^vii^	3.566 (3)	H7B···O2^viii^	2.8600
			
O1—N1—O2	123.57 (18)	C2—C1—H1	120.00
O1—N1—C3	118.53 (19)	C6—C1—H1	120.00
O2—N1—C3	117.90 (18)	C1—C2—H2	121.00
C2—C1—C6	120.7 (2)	C3—C2—H2	121.00
C1—C2—C3	117.56 (18)	C3—C4—H4	120.00
N1—C3—C2	118.37 (17)	C5—C4—H4	120.00
N1—C3—C4	118.69 (17)	C1—C6—H6	120.00
C2—C3—C4	122.94 (16)	C5—C6—H6	120.00
C3—C4—C5	119.12 (18)	Cl1—C7—H7A	109.00
C4—C5—C6	118.70 (17)	Cl1—C7—H7B	109.00
C4—C5—C7	120.40 (18)	C5—C7—H7A	109.00
C6—C5—C7	120.90 (17)	C5—C7—H7B	109.00
C1—C6—C5	120.98 (17)	H7A—C7—H7B	108.00
Cl1—C7—C5	110.94 (15)		
			
O1—N1—C3—C2	8.1 (3)	N1—C3—C4—C5	179.84 (18)
O1—N1—C3—C4	−171.9 (2)	C2—C3—C4—C5	−0.1 (3)
O2—N1—C3—C2	−171.8 (2)	C3—C4—C5—C6	0.4 (3)
O2—N1—C3—C4	8.3 (3)	C3—C4—C5—C7	−179.86 (18)
C6—C1—C2—C3	−0.1 (3)	C4—C5—C6—C1	−0.5 (3)
C2—C1—C6—C5	0.4 (3)	C7—C5—C6—C1	179.7 (2)
C1—C2—C3—N1	180.0 (2)	C4—C5—C7—Cl1	81.5 (2)
C1—C2—C3—C4	−0.1 (3)	C6—C5—C7—Cl1	−98.7 (2)

Symmetry codes: (i) −*x*+1, *y*+1/2, −*z*+1/2; (ii) −*x*+2, −*y*+2, −*z*+1; (iii) −*x*+2, −*y*+3, −*z*+1; (iv) *x*, −*y*+3/2, *z*+1/2; (v) *x*, −*y*+5/2, *z*+1/2; (vi) *x*, *y*+1, *z*; (vii) *x*, *y*−1, *z*; (viii) *x*, −*y*+3/2, *z*−1/2; (ix) −*x*+2, *y*−1/2, −*z*+1/2; (x) −*x*+2, *y*+1/2, −*z*+1/2; (xi) −*x*+1, *y*−1/2, −*z*+1/2; (xii) *x*, −*y*+5/2, *z*−1/2.

### Hydrogen-bond geometry (Å, °)

**Table:**

*D*—H···*A*	*D*—H	H···*A*	*D*···*A*	*D*—H···*A*
C2—H2···O1^iii^	0.93	2.67	3.583 (3)	166
C6—H6···O2^viii^	0.93	2.67	3.374 (3)	133

Symmetry codes: (iii) −*x*+2, −*y*+3, −*z*+1; (viii) *x*, −*y*+3/2, *z*−1/2.

## References

[bb1] Allen, F. H., Kennard, O., Watson, D. G., Brammer, L., Orpen, A. G. & Taylor, R. (1987). *J. Chem. Soc. Perkin Trans. 2*, pp. S1–19.

[bb2] Bruker (2007). *APEX2* and *SAINT* Bruker AXS Inc., Madison, Wisconsin, USA.

[bb3] Farrugia, L. J. (1997). *J. Appl. Cryst.* **30**, 565.

[bb4] Farrugia, L. J. (1999). *J. Appl. Cryst.* **32**, 837–838.

[bb5] Livermore, A. H. & Sealock, R. R. (1947). *J. Biol. Chem.* **167**, 699–704. 20287900

[bb6] Moreno, S. N. J., Schreiber, J. & Mason, R. P. (1986). *J. Biol. Chem.* **261**, 7811–7815. 3011800

[bb7] Sheldrick, G. M. (2008). *Acta Cryst.* A**64**, 112–122. 10.1107/S0108767307043930 18156677

[bb8] Spek, A. L. (2009). *Acta Cryst.* D**65**, 148–155. 10.1107/S090744490804362X PMC2631630 19171970

